# Evaluating Forelimb and Hindlimb Joint Conformation of Morna Racehorses (*Equus caballus*)

**DOI:** 10.3390/vetsci12010020

**Published:** 2025-01-05

**Authors:** Israr Ahmad, Sahar Ijaz, Mirza M. Usman, Ayesha Safdar, Imdad U. Khan, Muhammad Zeeshan, Syed S. U. H. Bukhari

**Affiliations:** 1 Department of Basic Veterinary Sciences, Faculty of Veterinary and Animal Sciences, Gomal University, Dera Ismail Khan 29220, Pakistan; israrahmad@gu.edu.pk; 2 Department of Anatomy and Histology, University of Veterinary and Animal Sciences, Lahore 54000, Pakistan; mirza.usman@uvas.edu.pk; 3 Department of Surgery, University of Veterinary and Animal Sciences, Lahore 54000, Pakistan; ayesha.safdar@uvas.edu.pk; 4 Department of Clinical Sciences, Faculty of Veterinary and Animal Sciences, Gomal University, Dera Ismail Khan 29220, Pakistan; imdadsaifi@gmail.com; 5 Jockey Club College of Veterinary Medicine and Life Sciences, City University of Hong Kong, Hong Kong SAR 523808, China; mzeeshan6-c@my.cityu.edu.hk

**Keywords:** body condition score, equine biomechanics, goniometer, horse: joint anatomy, joint conformation, limb joint angles, measuring joint angles, Morna breed, racehorses

## Abstract

Measuring limb joint angles is crucial for understanding horse conformation, performance, injury diagnosis, and prevention. While Thoroughbred horses have been extensively studied, local Pakistani breeds (e.g., Morna racehorse) have not received scientific attention. This study aimed to quantify normal angles of limb joints in the Morna breed. Limb joint angles of standing horses (n = 50) were quantified using a measuring tape, height stick, protractor scale, and goniometer. The mean and standard deviation (Mean ± SD) values for the forelimb joint angles were 123.02 ± 3.46° for elbow, 171.52 ± 2.39° for knee, and 147.68 ± 5.11° for fetlock. The mean ± SD values for the hindlimb joint angles were 128.62 ± 4.08° for stifle, 160.40 ± 3.89° for hock, and 155.48 ± 2.68° for fetlock. There was a non-significant (*p* > 0.05) correlation between horse joint angles and, age, body weight, and body condition score (BCS). The elbow and stifle joint angles of Morna align well with characteristics associated with optimal racing performance. However, the hock and fetlock angles differ from jumping Thoroughbred and French trotters. We recommend further research to examine the conformation of the Morna breed, particularly by measuring the lengths of limb segments and correlating it with joint angles. This may provide valuable insights into individual variations within the breed.

## Simple Summary

The assessment of limb joint angles is critical for understanding horse conformation, performance, and safety. This study aimed to determine the normal limb joint angles of the Morna breed, a Pakistani racehorse that has received little academic attention. We used standard techniques to measure joint angles of the forelimbs and hindlimbs. Morna racehorses had elbow and stifle joint angles correlated with peak racing performance, but their hock and fetlock angles differed from other breeds, for example, jumping Thoroughbreds and French trotters. This underscores the necessity for additional research into the Morna breed’s conformation, such as measuring limb segment lengths and comparing them to joint angles. Overall, quantifying joint angles in horses, especially in understudied breeds such as the Morna racehorses, is critical for understanding conformation, performance, and injury prevention.

## 1. Introduction

Horseracing is an ancient sport that has evolved alongside human civilization [1]. Successful horseracing requires horses with high-speed running abilities, largely determined by each horse’s conformation and limb soundness [2]. It is common for racehorses to develop soundness issues at a young age, often due to poor skeletal conformation [3]. In racehorses, the most likely anatomical locations for injuries can often be predicted based on the horse’s conformation [2]. Several factors influence a horse’s conformation, including genetics, racing surface, prior injuries or pathologies, biomechanics, and the horse’s age [3]. Horse stride problems occur due to anatomically compromised limbs, such as gait asymmetry, and unstable gait [4,5]. To assess whether an athletic horse is likely to succeed or fail, it is essential to evaluate its conformation in relation to speed [6]. Biomechanical limitations are associated with equine musculoskeletal disorders, such as angular limb deformities. Key conformational traits of the limbs include leg stance, hoof quality, and joint angles [6].

Limbs play a crucial role in movement efficiency and performance in race events. The structure and alignment of the leg bones directly influence the horse’s function [6]. Racehorses, in particular, place extra strain on their hindlimbs, relying on their hindquarters for engagement and collection [7,8]. Amid higher activity in the hindlimbs and the pelvic area, the soft tissues, ligaments, and joints are at a higher risk for injuries [7,8,9]. Additionally, the forelimbs experience substantial load during take-off and landing, carrying the horse’s total weight upon landing [10]. Consequently, the distal limb joints and surrounding soft tissues face extra stress and weight [11]. The rate of progression is directly linked to the health of the musculoskeletal system [2]. Poor conformation often indicates defects in the tendons and ligaments, leading to improper joint angles [12].

Measuring the angles of limb joints is a crucial aspect of studying horse conformation, as it plays a significant role in evaluating performance and selecting a high-performance racehorse [13,14]. It also helps prevent injuries, diagnose lameness, monitor training and rehabilitation, and make informed breeding and horse selection decisions [2,15,16,17]. Understanding limb joint angles provides valuable insights into a horse’s musculoskeletal health, enabling proactive management strategies that ensure optimal performance, reduce the risk of injuries, and promote long-term health and welfare [14,18,19,20].

Conformation evaluation is subjective and relies on the assessor’s experience, which can vary among experts [14,20,21]. For the most accurate assessment, a horse’s limb conformation should be evaluated while it stands straight on a hard, level surface under the observation of an experienced coach [16]. Every region has its evaluation procedures based on specific standards and breed-specific laws. While extensive research was conducted on angulation and conformation in long-distance racehorse breeds [14,16,20], local and short-distance Pakistani racehorses have received little attention.

This study aimed to evaluate and report normal angles of limb joints in Pakistan’s Morna breed of racehorses and establish baseline measurements for joint conformation. We hypothesized that documenting normal angles in healthy horses will create standard benchmarks for Morna racehorses and assist local researchers in conducting further biomechanical studies on this lesser-known horse breed.

## 2. Materials and Methods

### 2.1. Study Area and Study Design

This study was conducted in southern Khyber Pakhtunkhwa, Pakistan, involving active Morna breed racehorses (four to six years of age) with a good body condition score (four to six out of nine). Data were collected from 50 male racehorses. Before the start of data collection, written consent was obtained from the horse owners. Horses with a history of bone fractures, chronic musculoskeletal diseases, or any musculoskeletal defects and medication were excluded from this study.

### 2.2. Measurements

**Figure 1:**
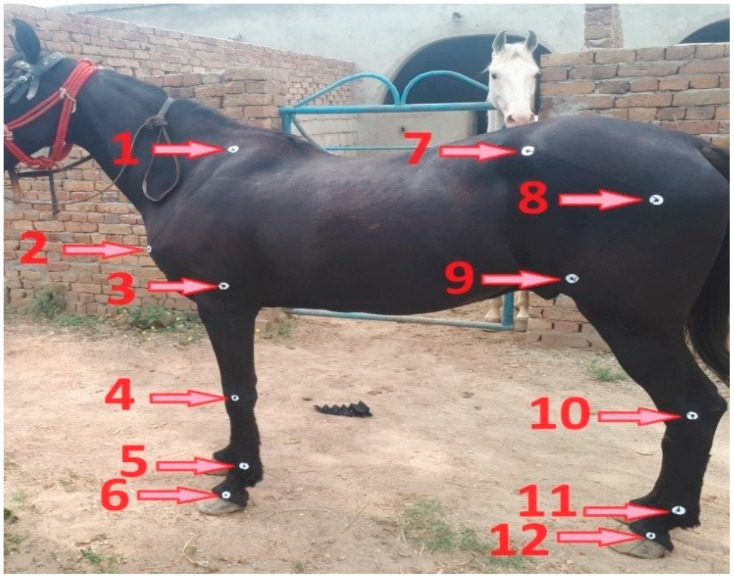
Standard joint positions and body reference points. The numbered reference points mentioned in the figure show the middle of the (1) dorsal border of the scapula, (2) point of the shoulder, (3) elbow joint, (4) knee joint, (5) fore fetlock joint, (6) fore pastern joint, (7) tuber coxae, (8) hip joint, (9) stifle joint, (10) hock joint, (11) hind fetlock joint, and (12) hind pastern joint.

Measurements were taken on racehorses while they stood in a normal position (Figure 1). We used a measuring tape, height stick, goniometer, and protractor scale and established predetermined reference points for accuracy [16], (Figure 1). Goniometry was used for angle measurement, as it is simple, practical, cost-effective, less labor-intensive, repeatable, and reliable [22]. This method does not require sophisticated equipment and is widely used in conformational studies [22,23,24]. This is particularly significant in low-resource settings where access to advanced technology may be limited. Before determining the joint angles, we established reference points based on their full range of motion to identify the axis of rotation for each joint. We selected easily palpable landmarks that had been used in previous conformational studies [16], (Figure 1). Data collection was conducted on the left side of the animals, and all the measurements were taken by a single researcher.

We quantified shoulder versus ground angle, shoulder joint angle, elbow joint angle, knee joint angle, fore fetlock joint angle, fore pastern versus ground angle, hip joint angle, stifle joint angle, hock joint angle, hind fetlock joint angle, and hind pastern versus ground angle according to existing studies [14,16]. The data on all angles was collected using a measuring tape, a height stick, a protractor scale, and a goniometer. The length of the goniometer’s arms was modified according to the researcher’s needs; for example, for carpal and tarsal measurements, the arms of the goniometer were extended, and for metacarpophalangeal and metatarsophalangeal measurements, the arms were shortened.

Body condition scores (BCS) of all horses were measured, and for weight determination, girth circumference was measured by measuring tape, and a height stick measured body length. The weight of the horse was measured with the help of the following formula [25]:

Horse body weight = ((Girth^2^ (cm) ×  Body length (cm))/2.54^3^/660 + 22.7

### 2.3. Data Collection

#### 2.3.1. Shoulder Versus Ground Angle

**Figure 2:**
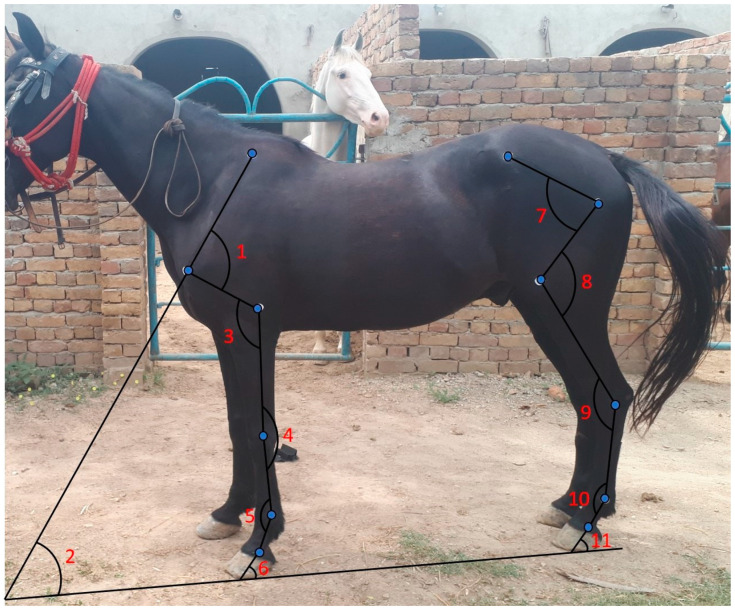
Studied angles: (1) shoulder joint angle; (2) shoulder versus ground angle; (3) elbow joint angle; (4) knee joint angle; (5) fore fetlock joint angle; (6) fore pastern versus ground angle; (7) hip joint angle; (8) stifle joint angle; (9) hock joint angle; (10) hind fetlock joint angle; and (11) hind pastern versus ground angle.

A straight line was drawn from the middle of the dorsal border of the scapula down to the point of the shoulder using a height stick. From the point of the shoulder, the line was extended distally and cranially toward the ground until it contacted the ground surface. Another straight line was drawn horizontally on the ground. The angle was measured by placing the protractor scale directly on the ground at the point where the lines from the shoulder and the ground intersect (Figure 2).

#### 2.3.2. Shoulder Joint Angle

A straight line was drawn from the middle of the dorsal border of the scapula down to the point of the shoulder using a height stick. Another straight line was drawn from the middle of the shoulder joint toward the middle of the elbow joint. A protractor was positioned at the shoulder joint, ensuring it was angled in relation to the line leading from the shoulder to the elbow, and the angle was measured with a goniometer. The disc of the goniometer was placed at the middle of the shoulder joint, with its fixed arm aligned along the spine of the scapula, while the movable arm extended toward the midline of the humerus, pointing toward the middle of the elbow joint (Figure 2).

#### 2.3.3. Elbow Joint Angle

A straight line was drawn from the middle of the shoulder joint to the middle of the elbow joint. Another straight line was drawn from the elbow joint to the middle of the knee joint using a height stick. A protractor was placed at the middle of the elbow joint, with one line extending toward the shoulder and the other toward the knee. The angle was measured using a goniometer, and the disc was placed at the axis of the elbow joint. One arm of the goniometer was aligned with the line from the shoulder to the elbow joint, while the other arm extended from the elbow to the knee joint (Figure 2).

#### 2.3.4. Knee Joint Angle

A straight line was drawn from the middle of the elbow joint to the middle of the knee joint. Another line extended from the middle of the knee joint to the middle of the fore fetlock joint. A protractor was placed obliquely at the knee joint. The angle was measured using a goniometer, with one arm positioned at the middle of the line extending from the elbow joint proximally and the other arm aligned with the line extending from the knee to the fore fetlock distally (Figure 2).

#### 2.3.5. Fore Fetlock Joint Angle

A straight line was drawn from the knee joint’s midpoint to the fore fetlock joint. Another line was drawn from the middle of the fore fetlock joint to the middle of the fore pastern joint. A protractor was placed over the fore fetlock joint, aligning it with the junction of the proximal and distal lines. The angle was measured using a goniometer, with one arm positioned proximally at the middle of the metacarpal bone and the other arm placed distally at the middle of the first phalanx (Figure 2).

#### 2.3.6. Fore Pastern Versus Ground Angle

A straight line was drawn from the fore pastern joint to the ground, extending distally and cranially. Another straight line was drawn horizontally at the leveled ground surface. The angle was measured by placing a protractor at the point where the line from the fore pastern intersects with the ground (Figure 2).

#### 2.3.7. Hip Joint Angle

A straight line was drawn from the middle of the tuber coxae to the middle of the hip joint. Another straight line extended from the middle of the hip joint toward the middle of the stifle joint. A protractor was placed obliquely at the hip joint, and the angle was measured using a goniometer, positioning its disc at the center of the hip joint with the fixed arm pointing toward the tuber coxae and the movable arm toward the center of the femur bone (Figure 2).

#### 2.3.8. Stifle Joint Angle

A straight line was drawn from the middle of the hip joint to the middle of the stifle joint. Another straight line was drawn from the middle of the stifle joint to the middle of the hock joint. A protractor was placed at the center of the stifle joint, and the angle was measured using a goniometer, with the disc positioned at the axis of the stifle joint. The fixed arm of the goniometer was aligned with the middle of the femur bone, while the other arm pointed towards the middle of the hock joint (Figure 2).

#### 2.3.9. Hock Joint Angle

A straight line was drawn from the middle of the stifle joint to the middle of the hock joint. Another straight line was drawn from the middle of the hock joint to the middle of the hind fetlock joint. A protractor was positioned obliquely at the hock joint, and the angle was measured using a goniometer, with one arm placed at the proximal end of the first line and the other arm at the distal end of the second line (Figure 2).

#### 2.3.10. Hind Fetlock Joint Angle

A straight line was drawn from the middle of the hock joint to the middle of the hind fetlock joint. Another straight line was drawn from the middle of the hind fetlock joint to the middle of the hind pastern joint. A protractor was placed at the hind fetlock joint, where both lines intersect, and the angle was measured using a goniometer, with one arm placed proximally at the first line and the second arm placed distally at the second line (Figure 2).

#### 2.3.11. Hind Pastern Versus Ground Angle

A straight line was drawn from the middle of the hind pastern joint, extending cranially to the midpoint of the hoof and continuing toward the ground. A second line was drawn horizontally on the leveled ground surface. The angle between the two lines was measured by placing the protractor at the point where the lines intersect (Figure 2).

#### 2.3.12. Girth Circumference and Body Length

The girth circumference was measured at the highest point of the withers and just behind the elbow and forelimb. This measurement was taken while the horse stood square at the level surface and at the moment of exhalation to ensure that additional lung volume was not included. Body length was measured from the point of the buttocks to the point of the shoulder.

### 2.4. Statistical Analysis

Age, girth circumference, BCS, body length, and weight are presented as mean and standard deviations (mean ± SD). All the studied angles are presented as box and violin plots in addition to mean ± SD. Data were tested for normality using the Shapiro–Wilk test. Horse joint angles and, age, body weight, and BCS were compared using Pearson correlation. Heatmaps were created based on the Pearson correlation coefficients (r) and *p*-values using R package ggplot2. All statistical analysis and data visualization were carried out using RStudio with R version 4.2.3 [26].

### 2.5. Ethical Approval

This study was approved by the Institutional Ethical Review Committee of the University of Veterinary and Animal Sciences Lahore, Pakistan (Approval reference no. DR/06).

## 3. Results

### 3.1. Body Measurements

The mean ± SD values for the age of the participating horses were 4.81 ± 0.67 years. The mean girth circumference and body length were 65.06 and 54.66 inches, respectively. The mean body weight was 350.42 kg, and the mean BCS was 4.90 out of 9 (Table 1).

### 3.2. Elbow, Knee, Fetlock, Stifle, and Hock Joint Angles

**Figure 3:**
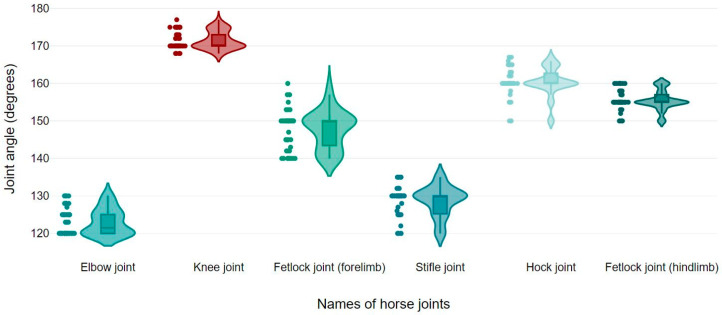
Box and violin plots for elbow, knee, fetlock (forelimb and hindlimb), stifle, and hock joint angles of the horses.

Mean ± SD values for elbow, knee, and fore fetlock joint angles were 123.02 ± 3.46°, 171.52 ± 2.39°, and 147.68 ± 5.11°, respectively. The mean ± SD values for stifle, hock, and hind fetlock joint angles were 128.62 ± 4.08°, 160.40 ± 3.89°, and 155.48 ± 2.68°, respectively (Figure 3).

### 3.3. Shoulder, Pastern, and Hip Joint Angles

**Figure 4:**
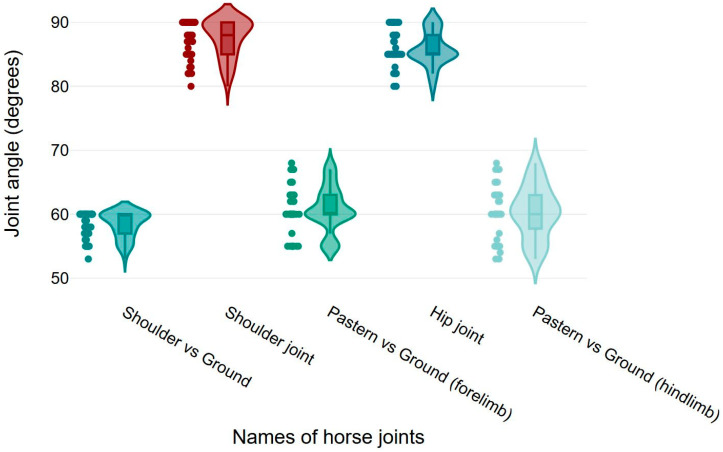
Box and violin plots for shoulder versus ground, shoulder, pastern versus ground (forelimb), pastern versus ground (hindlimb), and hip joint angles of the horses.

Mean ± SD values for shoulder and hip joint angles were 87.57 ± 2.94° and 85.82 ± 2.62°, respectively. The mean ± SD values for shoulder versus ground, fore pastern versus ground, and hind pastern versus ground angles were 58.38 ± 2.02°, 60.80 ± 3.53°, and 60.28 ± 4.01°, respectively (Figure 4).

### 3.4. Correlation Between Horse Joint Angles, Age, Body Weight, and Body Condition Score

**Figure 5:**
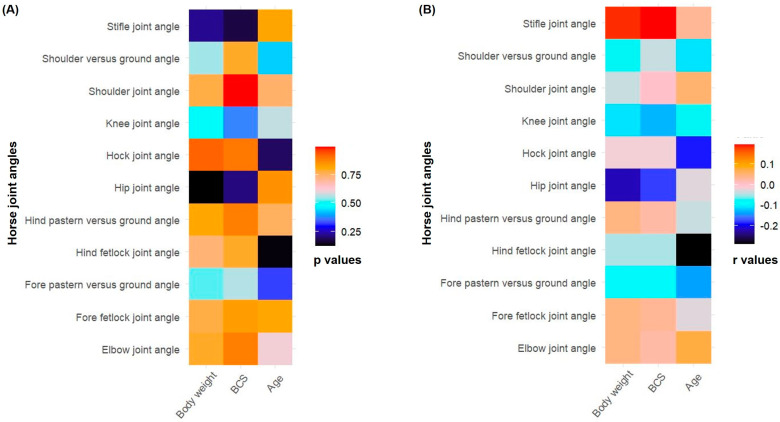
Heatmaps showing Pearson correlation between horse joint angles and age, body weight, and body condition score (BCS): (**A**) heatmap with respect to Pearson correlation *p*-values; (**B**) heatmap with respect to Pearson correlation coefficients (r values).

Non-significant correlations (*p* > 0.05) were found between horse joint angles and, age, body weight, and BCS. The Pearson correlation coefficients (r) and *p*-values between joint angles and, age, body weight, and BCS are presented (Figure 5).

## 4. Discussion

This study aimed to evaluate and report normal limb joint angles and establish baseline measurements for joint conformation in the Morna breed of racehorses. The conformational assessment serves as a valuable indicator of soundness, aiding in the selection process to minimize the risk of lameness and identify horses with optimal racing potential [20]. Several methods for measuring joint angles are available, including goniometry, radiography, and advanced digital imaging techniques [22,24]. Among these, goniometry is important as it is simple, practical, cost-effective, less labor-intensive, repeatable, and reliable [22]. This method does not require sophisticated equipment and is widely used in conformational studies [22,23,24]. This is particularly significant in low-resource settings where access to advanced technology may be limited. We measured girth circumference, body length, weight, BCS, forelimbs, and hindlimb joint angles. The elbow joint and pastern versus ground angles in Morna racehorses show promising alignment with characteristics associated with optimal racing performance. However, further assessment and consideration of potential breed-specific variations would contribute to a more comprehensive understanding of their individual suitability and selection for racing and show jumping.

The correlations between horse joint angles and, age, body weight, and BCS were not significant. Currently, there are no studies directly comparing limb joint angles with age, body weight, or BCS. However, in mature horses, the angles of the metacarpophalangeal and distal interphalangeal joints remain relatively constant [27]. This may explain the non-significant correlation observed in Morna racehorses aged four to five years. Additionally, horse age is a significant predictor of radiographic changes in joints, with scores increasing by 0.2 points per year [28]. Interestingly, in beef cows, BCS was positively correlated with foot angle scores [29], likely due to the increased body weight associated with a higher BCS. Moreover, morphometric measurements, including limb lengths and joint angles, were associated with musculoskeletal disorders in jumping Thoroughbreds [30]. This underscores the need for further research on horse limb joint angles in relation to racing and associated skeletal malfunctions.

The shoulder joint angle of 87.56° in Morna racehorses is comparable to the findings in elite show jumping horses [31]. However, the recorded shoulder joint angle was not similar to previous studies conducted on jumping Thoroughbreds (99°) [14] and racing French trotters (116°) [16], respectively. This may be due to the varying methods used for measurement in different studies and the breed-specific differences. Moreover, differences in angle measurements may arise from errors in angular data due to skin displacement across anatomical landmarks, particularly in the proximal limb [32]. The elbow joint angle was 123.02° in Morna racehorses, consistent with the finding (124°) in racing French trotters [16]. While differing from the findings (138°) in jumping Thoroughbreds [14]. This difference may be attributed to breed-specific variations in conformation. The angle of the elbow joint is particularly important when analyzing the unique traits of French trotters in their racing performance, as even minor changes in the elbow angle can have a huge impact on the outcome of a race [16]. Moreover, the elbow joint of horses is crucial for stride length and overall movement fluidity, particularly in activities that require agility and speed [33]. A horse’s potential increases with a lower elbow joint angle, and 122° is considered ideal elbow angle in French trotters [16]. In our study, the recorded elbow angle of 123.02° in Morna racehorses is close to the ideal value suggested by the previous study in French trotters [16]. Understanding these variations is crucial for breeders, trainers, and buyers to avoid potential limitations in athletic potential due to conformational defects.

The knee joint angle of 171.52° in Morna racehorses was similar to the angle observed in Swedish warmblood [31] and jumping Thoroughbred horses [14]. The optimal value of this angle should be 180° in racing French trotters [16]. The moderate variability, reflected in a standard deviation of 2.39° in Morna racehorses, underscores the need for individualized assessment when evaluating horses for races. The fore fetlock joint angle of 147.68° was seen in our study, whereas angles of 149° and 142° were seen in French trotters [16] and jumping Thoroughbreds [14], respectively. A higher fore fetlock joint angle increases the race potential of a horse, and 156° is considered an ideal value in racing French trotters [16].

The hip joint angle was 85.82° for Morna racehorses. Meanwhile, angles reported in studies conducted on Thoroughbred horses (88°) and racing French trotters (99°) were higher. Skin displacement at anatomical landmarks can lead to errors in angular measurements [32]. Interesting interbreed variations may originate from different degrees of skin movement rather than true variability in skeletal mobility. This may explain the slight angle difference between our and previous studies measuring joint angles. A recent study suggests that a horse’s potential for racing performance increases with the decrease in hip angle, and an ideal angle value is 90° [16].

The stifle angle in our study was 128.62°, which is greater than the angle (114°) reported in jumping Thoroughbred horses [14]. A higher stifle angle increases the potential of a racehorse [16]. The hock angle of 160.40° in Morna racehorses is comparable to Swedish warmblood horses [31]. However, our results are inconsistent with the findings in jumping Thoroughbred horses (148°) and French trotters (142°) respectively [14,16,20]. This may be because of interbreed differences in joint angles. Furthermore, the ideal value of hock angle is 142° in French trotters, and a decrease in stifle angle increases the race potential of a horse [16].

The hind fetlock angle was 155.48°, consistent with the findings (157°) in jumping Thoroughbred horses [14]. However, our results are inconsistent with the findings (142°) in French trotters [16]. Furthermore, it is reported that the optimal value of the hind fetlock angle is 142°, and increasing the hind fetlock angle increases the race potential of a horse [16]. In summary, the hip angle, stifle angle, and the angle of the pastern in relation to the ground in Morna racehorses exhibit promising alignment with characteristics associated with optimal racing performance. However, the angle of hock and fetlock differ from those found in previous studies of jumping Thoroughbreds and French trotters. It is important to note that we had not calculated the sample size or conducted a power analysis, which may present a potential limitation of this study. We recommend conducting further research to explore the conformation of this specific breed using advanced techniques and equipment, such as modern imaging methods and artificial intelligence software, for example, Quintic Biomechanics video analysis software. Special emphasis should be placed on measuring the lengths of limb segments and correlating these measurements with the angles. This approach may provide valuable insights into individual variations within the breed.

## 5. Conclusions

Our study offered valuable insights into the conformational features of Morna racehorses. The joint angles and body measurements we observed provide a foundation for creating a database for selecting Morna racehorses, considering their unique traits. Breeders, trainers, and buyers need to understand the breed-specific variations discussed in our study to make informed decisions about the athletic potential and performance capabilities of individual Morna racehorses. Additional research should focus on examining the conformation of the Morna breed, particularly by measuring the lengths of limb segments and correlating them with joint angles. This investigation may reveal further insights into individual variations within the breed.

## Figures and Tables

**Table 1:** Mean and standard deviation (SD) values for age, girth circumference, body condition score (BCS), body length, and weight.

Measurements	Mean	SD
Age (years)	4.81	0.67
Girth Circumference (inch)	65.06	0.91
Body Length (inch)	54.66	1.02
Body Weight (kg)	350.42	15.56
BCS (/9)	4.90	0.81

## Data Availability

The supporting raw data of this research will be made available upon request.
